# Effects of Taekwondo Training on Growth Factors in Normal Korean Children and Adolescents: A Systematic Review and Meta-Analysis of Randomized Controlled Trials

**DOI:** 10.3390/children10020326

**Published:** 2023-02-08

**Authors:** Guyeol Jeong, Hongyong Jung, Wi-Young So, Buongo Chun

**Affiliations:** 1 Social Physical Education, Sunchon National University, Sunchon 57922, Republic of Korea; 2 Department of Taekwondo, Art, Design, & Physical Education, Chosun University, Gwangju 61452, Republic of Korea; 3 Sports Medicine Major, College of Humanities and Arts, Korea National University of Transportation, Chungju-si 27469, Republic of Korea; 4 Graduate School of Physical Education, Myongji University, Yongin 17058, Republic of Korea

**Keywords:** growth hormone, insulin-like growth factor-1, height, taekwondo exercise

## Abstract

The growth of children and adolescents is both an important health indicator and a major public health issue. Many recent studies have investigated the effects of taekwondo on growth factors, but no consensus has yet been reached. This meta-analysis aimed to determine the effects of taekwondo on the growth factors in children and adolescents (aged 8 to 16 years). Randomized controlled trials from PubMed, Web of Science, Cochrane Library, the Research Information Sharing Service, the Korea Citation Index, and the Korean-studies Information Service System were analyzed. The effect sizes (standardized mean differences, SMD) were calculated, the risk of bias and publication bias were assessed, and the effect size and subgroup analyses were pooled. We found that the taekwondo group had significantly higher levels of growth hormones (SMD 1.78, 95% confidence interval [CI] 0.98–2.58, and *p* < 0.001) and insulin-like growth factors (SMD 1.76, 95% CI 0.60–2.92, and *p* < 0.001) than the control group. For height, a medium effect size was observed (SMD 0.62, 95% CI −0.56–1.80, and *p* = 0.300), but the between-group difference was not significant. Thus, taekwondo had significant positive effects on the secretion of growth hormones and insulin-like growth factors in Korean children and adolescents. A longitudinal follow-up is necessary to determine the effect on height. This suggests that taekwondo can be recommended as an appropriate physical exercise for maintaining normal growth in children and adolescents.

## 1. Introduction

Taekwondo, a sport that originated in Korea, has been growing in popularity worldwide since its adoption as an official Olympic sport in 2000 [1]. Currently, over 80 million people from 209 countries practice taekwondo, and the number of children learning taekwondo is increasing annually [2,3]. South Korea (hereinafter Korea) alone houses approximately 9709 taekwondo studios [4], in which training programs are provided mostly for children and adolescents to improve their health and physical strength and build their character [5]. Hence, several studies have examined the effects of taekwondo on children and adolescents in Korea. The main foci of these studies were the physiological effects of taekwondo [6,7,8] and the injuries sustained during sparring [9,10,11,12].

As the body of evidence grows, studies are focusing on synthesizing and analyzing the physical effects of taekwondo. In a synthesizing analysis of the effects of taekwondo on children’s physical fitness factors, Nam and Lim [5] reported that taekwondo improved cardiorespiratory and muscular endurance as well as strength, which are important physical abilities in children. According to a previous study, taekwondo can promote physical growth by developing physical strength and coordination [13]. Growth is comprised of a series of developmental stages over a period of approximately 20 years, including the fetal period [14]. The normal growth of children and adolescents is a key health indicator and is considered an important public health issue [15]. Therefore, analyzing the effects of physical exercise conducive to normal growth is an important contribution to public health.

Growth refers to an increase in the size of all or a part of the body. Nutrition and physical exercise are important for the normal growth of children and adolescents aged 8 to 16 years; in particular, physical exercise is involved in stimulating the secretion of growth-related hormones [16,17]. Changes in play-time behaviors of children and adolescents due to increasing smartphone usage and TV-related screen time are becoming growing concerns [18]. Moreover, infectious diseases, such as the coronavirus disease of 2019, have further limited the physical exercise performed by children and adolescents [19]. The World Health Organization [20] recommends that children and adolescents should engage in moderate to vigorous physical exercise for at least 1 h per day. Several studies have shown that there is a positive association between the level of physical exercise and growth [3,13,15].

The growth hormone (GH), which is secreted from the pituitary gland, induces growth of the epiphyseal region of long bones and is closely associated with the growth of children; it increases the absorption of amino acids into muscle proteins and plays an important role in lipolysis, sexual maturation, immunity, and metabolic regulation [21]. Physical exercise enhances children’s growth by inactivating somatostatin, which suppresses the secretion of GH, and thus, allows GHs to be secreted freely [22]. In addition, by stimulating the secretion of insulin-like growth factor-1 (IGF-1) in growing children, physical exercise plays a vital role in protein synthesis and cell proliferation, which stimulates growth. IGF-1 also increases the neurotransmitter levels [16,23].

On the other hand, obesity is known to have a negative effect on growth and development. The ability to secrete growth hormone is significantly reduced in the obese population compared to the normal-weight population of the same age. According to previous studies, the higher the level of obesity, the less periodic secretion of growth hormones during the circadian cycle, as the increase in free fatty acid in the blood inhibits the secretion of growth hormones [24,25]. In contrast, a decrease in free fatty acids in the blood increases the secretion of growth hormones. Therefore, as obesity increases, the secretion of growth hormones decreases, and thus, the ability of growth hormones to break down body fat decreases, which causes a negative feedback loop that inhibits growth and development [17]. However, it is said that if obese people lose weight through physical exercise and nutrition control, the secretion of growth hormones can be restored [17,25].

Physical exercise increases the amount and frequency of hormone secretion by directly stimulating the secretion of GHs, and the concentration of IGF-1 increases in proportion to the increase in GHs [26,27]. In particular, the axial activity of GH-IGF-1 is positively correlated with improvement in cardiorespiratory endurance and muscle mass [28]. Many different taekwondo movements stimulate the muscles and joints of the whole body, which improves cardiorespiratory endurance and muscle strength, and induces the release of growth factors [29,30]. However, despite the recent increase in the number of studies investigating the association between taekwondo and growth [29,30,31,32,33,34,35,36,37,38,39,40], there is no consensus regarding the effects of taekwondo on growth factors in children and adolescents. Therefore, the purpose of this systematic review and meta-analysis was to examine the effects of taekwondo interventions on children’s and adolescent’s growth-related factors. This study could provide a comprehensive rationale for a taekwondo intervention method that helps to induce normal growth, which is one of the main health indicators for children and adolescents [15].

## 2. Materials and Methods

### 2.1. Study Design

This systematic literature review, including the selection of the included articles, was carried out in accordance with the Preferred Reporting Items for Systematic Reviews and Meta-Analyses (PRISMA) reporting guidelines for systematic review and meta-analysis [41]. In addition, two researchers conducted the review independently. Our research program has been registered on Prospero, the International Register of Expectations for System Evaluation, with registration number CRD42023393490.

### 2.2. Literature Search Strategy

A literature search using 6 databases, from 3 English electronic databases (PubMed, Web of Science, and Cochrane Library), and 3 Korean databases (the Research Information Sharing Service [RISS], Korea Citation Index [KCI], and Korean-Studies Information Service System [KISS]) using the following search terms: taekwondo AND growth OR growth factor OR body composition OR height OR physique OR growth hormone OR hormone.

### 2.3. Literature Selection and Quality Assessment

For the literature analysis, the following selection criteria were applied: (1) studies on children aged 8 to 16 years with taekwondo experience; (2) studies on taekwondo exercise intervention; (3) studies including growth-related dependent variables, height, and growth-related hormones (GHs and IGF-1); and (4) randomized controlled trials (RCTs). The exclusion criteria were as follows: (1) studies on taekwondo athletes (i.e., competitive players); (2) studies including adult subjects; (3) case studies, comparative studies, and literature reviews; (4) studies unrelated to growth-related variables such as body fat content and blood lipid concentration; (5) studies not available in a full-text form; and (6) studies without clear statistical measures.

The quality of the selected literature was evaluated using the seven items of the Cochrane risk of bias (ROB) tool of the Review Manager program (RevMan 5.4, Cochrane Collaboration, Oxford, UK). The specific evaluation items are as follows: (a) random sequence generation (selection bias), (b) allocation concealment (selection bias), (c) blinding of participants and personnel (performance bias), (d) blinding of outcome assessment (detection bias), (e) incomplete outcome data (attrition bias), (f) selective outcome reporting (reporting bias), and (g) other sources of biases. Items were rated as either low risk, unclear, or high risk of bias. The overall risk of bias (ROB) is ranked by three levels according to the number of evaluation items that are rated as high risk of bias in the experiment: high risk (5 or more), medium risk (3 or 4), and low risk (2 or less). Two researchers separately performed the quality assessment, rating the seven items in terms of low ROB (+), unclear ROB (?), and high ROB (−). In case of disagreement, consensus was reached through discussion.

### 2.4. Data Analysis

The meta-analysis was performed using the Review Manager program (RevMan 5.4, Cochrane Collaboration, Oxford, UK). As the effect size, the mean difference (MD), or standardized mean difference (SMD) was selected for the same outcome variable. Statistical heterogeneity of the pooled studies was assessed using Cochrane’s Q test and the I^2^ statistic. A value of I^2^ between 50% and 90% possibly represents significant heterogeneity. When heterogeneity was detected among studies (Q statistic *p* < 0.1 or I^2^ statistic > 50%), a random-effects model was used, and when heterogeneity was not detected, a fixed-effect model was used [41]. In case of heterogeneity, the cause of heterogeneity was confirmed through sensitivity analysis. In addition, a subgroup analysis was performed to analyze whether differences in study subjects could explain the heterogeneity of the study, such as normal and obesity subjects. A funnel plot was employed to assess publication bias [42].

## 3. Results

### 3.1. Study Selection and Quality Assessment

**Figure 1:**
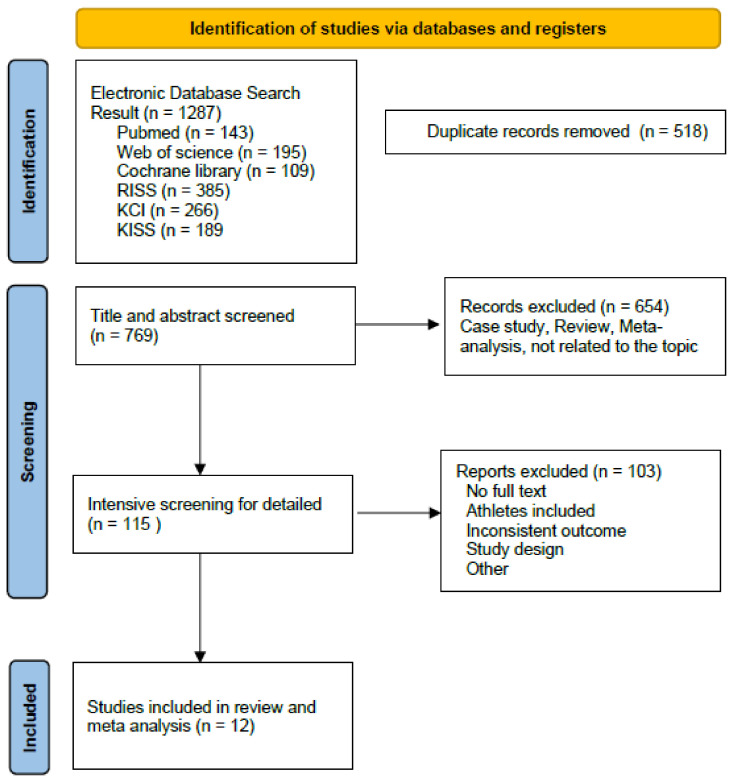
Flowchart of the study selection process.

A total of 1287 studies were retrieved in an initial search in the electronic databases (PubMed, n = 143; Web of Science, n = 195; Cochrane Library, n = 109; RISS, n = 385; KCI, n = 266; and KISS, n = 189). After removing 518 duplicates, 769 studies were retained. A total of 654 studies were then removed after screening of the titles and abstracts. Following an intensive screening process of the remaining 115 articles, 103 were excluded because they met the exclusion criteria (full text was not accessible, no controls, inclusion of athletes, inconsistent outcome indicators, and unclear results). Thus, a total of twelve studies that met all the inclusion criteria were selected for the final analysis (Figure 1).

### 3.2. Characteristics of the Selected Studies and Quality Assessment

Table 1 presents the characteristics of the selected studies. All twelve selected studies were randomized controlled trials. All studies were published between 2006 and 2018.

**Figure 2:**
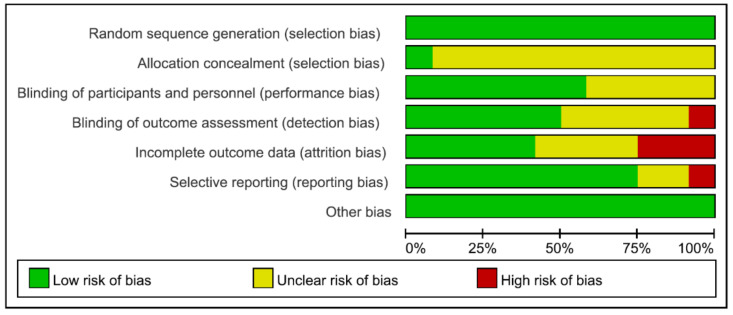
Risk of bias graph.

The majority of the selected studies were conducted in Korea. Notably, many non-Korean studies included taekwondo athletes. These studies were excluded according to the selection criteria [6,7,8,9,10,11,12]. A total of 260 children and adolescents were analyzed. One hundred and thirty people were trained in the taekwondo group (50.0%), and 130 people were in the control group (50.0%). The participants from the selected studies were 8–13 years old in ten studies (n = 214, 82.3%) and 14–16 years old in two studies (n = 46, 17.7%) [30]. Five studies examined obese children [30,31,32,33,37], and seven examined normal-weight children [29,34,35,36,38,39,40]. The taekwondo exercise intervention period was eight weeks (n = 2, age 11.75 ± 0.62–11.82 ± 0.65 years) [31,37], 12 weeks (n = 8, ages 11.1 ± 0.3–15.1 ± 0.88 years), or 16 weeks (n = 2, ages 11.20 ± 0.77–11.53 ± 0.64 years). The weekly frequency of the taekwondo exercise intervention was five days in eight studies, three days in three, and one day in one study. The taekwondo exercise time varied from 50 to 100 min per session. The items used as indicators of growth were height (n = 8), GHs (n = 8), and IGF-1 (n = 7). GHs and IGF-1 were evaluated from blood samples collected from the antecubital vein. All the studies’ control groups did not participate in regular physical exercise other than physical education classes at school. In the quality assessment of the selected literature using the seven items of the Cochrane risk of bias (ROB) tool, the “random sequence generation” and “blinding of participants and personnel” domains were rated to have a low risk of bias, the “allocation concealment” domain an unclear risk of bias in most studies, and the “incomplete outcome data” and “selective reporting” domains an unclear risk of bias in most studies and a high risk of bias in one or two studies (Figure 2).

## 4. Effects of Taekwondo on Growth

### 4.1. Effect on GH

**Figure 3:**
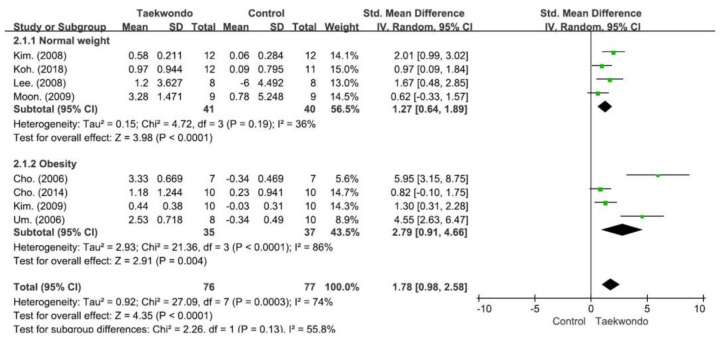
Forest plot depicting differences in the change-from-baseline growth hormone level between the taekwondo and control groups. CI: confidence interval and SD: standard deviation. (Kim. 2008 [29]; Koh. 2018 [34]; Lee. 2008 [39]; Moon. 2009 [35]; Cho. 2006 [31]; Cho. 2014 [32]; Kim. 2009 [30]; Um. 2006 [37]).

As shown in Figure 3, in a meta-analysis investigating whether taekwondo practice had an effect on the increase in the secretion of GHs in students, a significantly larger effect size on the secretion of GHs (SMD 1.78, 95% CI 0.98–2.58, and *p* < 0.001) was noted in the taekwondo group compared to the control group. With heterogeneity among the research results calculated at I^2^ = 74% (Chi^2^ = 27.09, *df* = 7, and *p* < 0.001), the selected studies were found to have interstudy heterogeneity. To evaluate the sensitivity of taekwondo to GHs, a meta-analysis was performed while iteratively removing one study after another. As a result of this leave-one-out sensitivity analysis, the studies by Um et al. [37] and Cho et al. [31] were suspected to have caused the interstudy heterogeneity. The meta-analysis performed after removing these two studies resulted in an SMD of 1.18, 95% CI of 0.76–1.59, I^2^ of 7%, and *p* of < 0.001 without a change in significance. As a result of the subgroup analysis, the taekwondo group showed a larger effect size than the control group in both the normal-weight and obese subgroups with a significant difference. In addition, there was no significant difference between the normal-weight subgroup and the obese subgroup (Chi^2^ = 2.26, *df* = 1, *p* = 0.13, and I^2^ = 55.8%).

### 4.2. Effect on the IGF-1

**Figure 4:**
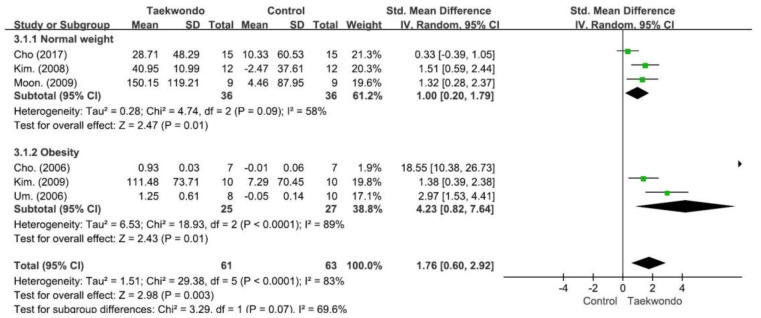
Forest plot depicting differences in the change-from-baseline insulin-like growth factor-1 levels between the taekwondo and control groups. CI: confidence interval and SD: standard deviation. (Cho. 2017 [38]; Moon. 2009 [35]; Kim.2008 [29]; Cho. 2006 [31]; Kim. 2009 [30]; Um. 2006 [37]).

As demonstrated in Figure 4, in a meta-analysis examining whether taekwondo affects the increase in the secretion of IGF-1 in students, a significantly larger effect size was noted on IGF-1 secretion (SMD 1.76, 95% CI 0.60–2.92, and *p* < 0.001) in the taekwondo group than in the control group. With heterogeneity among the research results calculated at I^2^ = 83.6% (Chi^2^ = 20.36, *df* = 4, and *p* < 0.01), the selected studies were found to have interstudy heterogeneity. In the leave-one-out sensitivity analysis performed to evaluate the sensitivity of taekwondo to IGF-1, the studies by Cho et al. [31], Cho et al. [38], and Um et al. [37] were suspected to have caused the interstudy heterogeneity. A meta-analysis of the remaining four studies resulted in an SMD of 1.42, 95% CI of 0.85–1.98, I^2^ of 0%, and *p* of <0.001, without a change in significance. As a result of the subgroup analysis, the taekwondo group was found to have a significantly larger effect size than the control group in both the normal-weight and obese subgroups. In addition, there was no significant difference between the normal-weight subgroup and the obese subgroup (Chi^2^ = 3.29, *df* = 1, *p* = 0.070, and I^2^ = 69.6%).

### 4.3. Effect on Height

**Figure 5:**
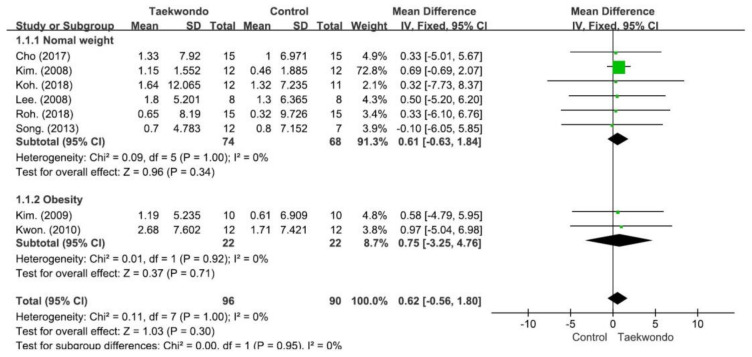
Forest plot depicting differences in the change-from-baseline height between the taekwondo and control groups. CI: confidence interval and SD: standard deviation. (Cho. 2017 [38]; Kim. 2008 [29]; Koh. 2018 [34]; Lee. 2008 [39]; Roh. 2018 [40]; Song. 2013 [36]; Kim. 2009 [30]; Kwon 2010 [33]).

As shown in Figure 5, in a meta-analysis examining the effects of taekwondo on height, a medium effect size on height (SMD 0.62, 95% CI −0.56–1.80, and *p* = 0.300) was noted in the taekwondo group compared with the control group, but it was without statistical significance. According to the subgroup analysis, this effect was evident in both the normal-weight and obese subgroups. With heterogeneity among the research results calculated at I^2^ = 0% (Chi^2^ = 0.11, df = 7, and *p* = 1.000), no interstudy heterogeneity was observed among the selected studies. In addition, there was no significant difference between the normal-weight subgroup and the obese subgroup (Chi^2^ = 0.00, *df* = 1, *p* = 0.950, and I^2^ = 0.0%).

### 4.4. Publication Bias Assessment

**Figure 6:**
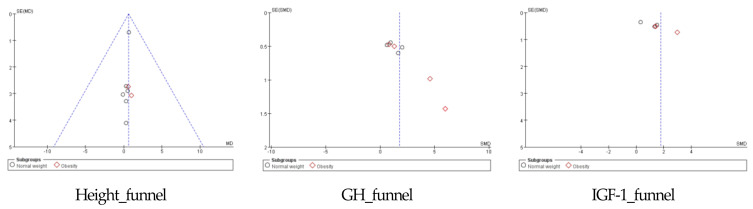
Funnel plot. GH: growth hormone and IGF-1: insulin-like growth factor-1.

A funnel plot analysis was performed to examine the publication bias of the selected studies, as shown in Figure 6. The funnel plot symmetry was good, with each variable appearing relatively symmetrical and evenly distributed. Taking all variables together, the publication bias was confirmed to be relatively low.

## 5. Discussion

This systematic literature review and meta-analysis was conducted to examine the effects of taekwondo on growth factors in children and adolescents. To this end, twelve RCTs were selected for the meta-analysis with a total of 260 children and adolescents aged 10 to 16 years in the taekwondo intervention group (n = 130) and control group (n = 130). Taekwondo practice was found to have beneficial effects by increasing the secretion of growth-related hormones (GH and IGF-1). In addition, these effects showed greater effects in children and adolescents with obesity. To the best of our knowledge, this is the first meta-analysis of studies investigating the effects of taekwondo on growth factors in children and adolescents. The results of this study suggest that taekwondo is an appropriate physical exercise intervention to promote the normal growth of children and adolescents.

A GH is an anterior pituitary hormone involved in protein synthesis and lipolysis and is responsible for bone and cartilage growth. Physical exercise has been reported to be a powerful physiological stimulus of GH secretion [43,44,45,46,47,48,49]. In our study, the taekwondo group showed a significant increase in the secretion of GHs compared to the control group. The exercise intensity during taekwondo practice reportedly ranged from 50 to 80% HRmax (rate of perceived exertion 13–16) [5]. Previous studies indicated that GHs rise at an exercise intensity of 30% of VO_2_ max and increases up to 100-fold in response to anaerobic exercise, depending on the type and intensity of exercise [50]. It has also been reported that exercise affects the secretion of GHs at night in addition to acute responses, thereby inducing an increase in the secretion of plasma levels at rest on a long-term basis [51,52]. The intensity and duration of taekwondo exercise stimulate and increase GH secretion, allowing the assumption that it may play a positive role in the physical development of school students. In addition, it meets the daily physical exercise intensity level and duration recommended by the WHO for the health of children and adolescents [20]. Therefore, taekwondo can be considered a recommended intervention for growth and health.

The growth hormone-insulin-like growth factor (IGF-I) axis is a major regulator of anabolic and muscle growth in the human body. In particular, it plays an important role in the physiological growth and development of childhood and adolescence [28,53,54,55,56,57,58,59,60,61,62,63,64]. Conditions that can interfere with the action of growth hormones in these childhood and adolescent populations can alter physiological growth and development, and negatively affect future health and well-being [21,65]. Obesity has been demonstrated to affect the metabolism and function of growth hormones through several biochemical pathways [24,25]. Although no significant difference was observed in GH secretion at rest in a related sub-group analysis, the effect tended to be greater in the obese group than in the normal-weight group. This may be attributable to the increase in GH secretion due to physical exercise duration and reduced body weight [24,25]. However, in the studies conducted by Cho et al. [32] and Um et al. [37], taekwondo intervention programs for obese adolescents were found to have a longer duration (90–100 min). Our finding is also supported by studies indicating that long-duration physical exercise has a positive effect on GH secretion [17,50]. In obese children, the frequency of GH secretion is reduced during the circadian cycle of GHs, such that their GH secretion is significantly lower than that of their normal-weight counterparts [66,67]. The results of our study suggest that the decreased GH secretion in the obese population can be normalized and enhanced by practicing taekwondo [68,69].

Key substances during the growth phase include growth-related anabolic hormones, particularly GHs and the IGF-1. GHs synthesizes and secretes IGF-1 by binding to GH receptors that are present in specific cells in the liver and skeletal system [66]. The synthesis and secretion of IGF-1 is stimulated by GHs. IGF-1 is known to promote growth by inducing the differentiation and proliferation of the growth plate chondrocytes of long bones. IGF-1 is dependent on GHs, and as the GH secretion increases, the IGF-1 concentration also increases [70]. Previous studies have suggested that physiological stimulation is required to increase the sensitivity of growth-related hormones, and physical exercise is an important physiological stimulator for the secretion of growth-related hormones in the body. Of note, it was reported that the secretion of growth-related hormones after physical exercise was high in childhood and adolescence [28,47,52]. Our meta-analysis revealed that the effect size, on increasing the concentration of the IGF-I, was larger in the taekwondo group that was exposed to a longer-duration intervention (12 weeks) than the control group. Further, as shown by the subgroup analysis, a larger trend of effect size was observed in obese students than in normal-weight students. This increase in the IGF-1 is attributable to an increase in GH secretion from the pituitary gland [71]. Previous studies yielded inconsistent results on the IGF-1 levels after physical exercise [72,73]. However, in light of the findings of a positive correlation between GH secretion, maximal oxygen uptake, and the IGF-1 levels [74,75], taekwondo is considered to be effective in inducing an increase in the IGF-1. That is, GH secretion that is increased by taekwondo stimulates the synthesis of the IGF-1, and physical strength enhanced by physical exercise has a positive effect on the synthesis of the IGF-1. Moreover, growth hormones and the IGF-1 affect metabolism-related actions in addition to growth-related actions. It is known that fat is used as an energy source to reduce body fat and increase lean body mass by acting on skeletal muscle and bones [66]. It is thought that the positive effect of this metabolism induces the desired growth and development of growing students. These results suggest that taekwondo elicits beneficial effects on growth by increasing the IGF-1 secretion in normal-weight and obese students.

Height growth is rapid during infancy followed by a stable growth rate in childhood, then it rapidly increases in adolescence, and finally slows down as one grows into adulthood. The rate of height growth is the highest in the first year of life and gradually decreases until the onset of pubertal growth spurts [76]. In our study, taekwondo was found to have a medium effect size on height, but without statistical significance, presumably due to the duration of the intervention of the studies included in the meta-analysis of this study. The intervention duration of the selected studies was either 8 (n = 2), 12 (n = 8), or 16 (n = 2) weeks. Children and adolescents are reported to grow 4–5 cm per year, excluding the peak height velocity [76,77,78,79,80,81]. Thus, it may be assumed that there was no significant difference in the height development in children and adolescents during the intervention period of 8–12 weeks. In this context, long-term observations (at least 1 year) are necessary to examine the effect of taekwondo on height [76]. On the other hand, although it was difficult to observe the short-term effect on height, taekwondo training is considered to be a sufficiently beneficial physical exercise for growth in students, because an increased secretion of internal growth-related hormones was observed. In addition, this beneficial effect was found to be greater in obese students. This suggests that obese students should be greatly advised to participate in taekwondo training.

### Limitations of the Study

This systematic review has a few limitations. First, most of the selected studies were conducted in Korea and the participants were mainly Koreans. Further research is needed to confirm the growth effect of taekwondo through the analysis on non-Koreans. Second, the randomized controlled trial showed relative publication trends, affecting the reliability of this systematic review and the findings. Future studies should use appropriate randomization, allocation concealment, blindfolding, and statistical methods to reduce the bias in the research design. Third, growth-related factors were evaluated only for the growth hormones, IGF-1, and height factors. If studies that additionally analyze other hormonal variables that evaluate future growth are reported, it is necessary to comprehensively analyze them. Fourth, maturation-related variables were not considered in the selected studies. Future studies should focus on analyzing maturation-related variables together. Lastly, growth hormones and the insulin-like-growth-factor-1 had high heterogeneity in the analysis results, even though similar measurement methods were used. Despite these limitations, this study presents results from a comprehensive analysis of the effects of a taekwondo exercise method for the growth of children and adolescents, providing valuable insight into taekwondo exercise’s role in normal growth and development.

## 6. Conclusions

In conclusion, taekwondo training significantly increased the GH and IGF-1 secretion in children and adolescents aged 10–16 years. This suggests that taekwondo can be recommended as an appropriate physical exercise for maintaining normal growth in children and adolescents.

## Figures and Tables

**Table 1:** Summary of the included studies.

First Author (Year)	Location (Language)	Participants	Study Design	Duration (Weeks)	Experimental Group (n) (Mean Age ± SD) Frequency/Week, Min/Session	Control Group (n) (Mean Age ± SD)	Outcomes
[29] Kim. (2008)	Korea (Korean)	12–13-year-old boys	RCT	12	Taekwondo (n = 12) (12.35 ± 0.48 years) 5 days/w, 70 min/session	Control (n = 12) (12.42 ± 0.50 years) No regular exercise Usual daily living	GH; IGF-1; and height
[30] Kim. (2009)	Korea (Korean)	13–16-year-old obese boys	RCT	12	Taekwondo (n = 10) (14.70 ± 1.25 years) 5 days/w, 50 min/session	Control (n = 10) (15.10 ± 0.88 years) No regular exercise Usual daily living	GH; IGF-1; and height
[31] Cho. (2006)	Korea (Korean)	9–12-year-old obese boys	RCT	8	Taekwondo (n = 7) (11.75 ± 0.62 years) 5 days/w, 90 min/session	Control (n = 7) (11.75 ± 0.62 years) No regular exercise Usual daily living	GH and IGF-1
[32] Cho. (2014)	Korea (Korean)	11–13-year-old obese boys	RCT	12	Taekwondo (n = 10) (11.77 ± 1.25 years) 3 days/w, 60 min/session	Control (n = 10) (11.1 ± 0.3 years) No regular exercise Usual daily living	GH
[33] Kwon. (2010)	Korea (Korean)	8–12-year-old obese girls and boys	RCT	12	Taekwondo (n = 12) (11.92 ± 0.90 years) 3 days/w, 60 min/session	Control (n = 12) (12.50 ± 0.80 years) No regular exercise Usual daily living	Height
[34] Koh. (2018)	Korea (Korean)	8–11 years girls and boys	RCT	12	Taekwondo (n = 12) 5 days/w, 50 min/session	Control (n = 11) No regular exercise Usual daily living	GH and height
[35] Moon. (2009)	Korea (Korean)	11–13-year-old girls	RCT	12	Taekwondo (n = 9) (12.18 ± 2.12 years) 5 days/w, 60 min/session	Control (n = 9) (12.73 ± 1.82 years) No regular exercise Usual daily living	GH; IGF-1 and height
[36] Song. (2013)	Korea (Korean)	13–14-year-old boys	RCT	12	Taekwondo (n = 12) (14.0 ± 0.64 years) 3 days/w, 50 min/session	Control (n = 10) (13.9 ± 0.46 years) No regular exercise Usual daily living	Height
[37] Um. (2006)	Korea (Korean)	9–12-year-old obese boys	RCT	8	Taekwondo (n = 8) (11.82 ± 0.65 years) 5 days/w, 100 min/session	Control (n = 11) (11.68 ± 0.59 years) No regular exercise Usual daily living	GH and IGF-1
[38] Cho. (2017)	Korea (English)	11–13-year-old girls and boys	RCT	16	Taekwondo (n = 15) (11.20 ± 0.77 years) 5 days/w, 60 min/session	Control (n = 15) (11.33 ± 0.72 years) No regular exercise Usual daily living	IGF-1 and height
[39] Lee. (2008)	Korea (Korean)	11-year-old boys	RCT	12	Taekwondo (n = 8) (11.1 ± 0.4 years) 5 days/w, 50 min/session	Control (n = 8) (11.1 ± 0.3 years) No regular exercise Usual daily living	GH and IGF-1
[40] Roh. (2018)	Korea (English)	11–13-year old girls and boys from multicultural families	RCT	16	Taekwondo (n = 15) (11.53 ± 0.64 years) 1 days/w, 60 min/session	Control (n = 15) (11.40 ± 0.63 years) No regular exercise Usual daily living	Height

RCT: randomized controlled trial; GH: growth hormone; IGF-1: insulin-like growth factor-1; w: week; SD: standard deviations). GHs and IGF-1 were analyzed from blood samples collected from the from the antecubital vein at baseline and after intervention.

## Data Availability

The data that support the findings of this study are available from the corresponding author, (B.C.), upon reasonable request.
